# Temporal Improvement in Outcomes of Surgical Treatment of Isolated Tricuspid Regurgitation in the United States

**DOI:** 10.1016/j.jacadv.2024.101319

**Published:** 2024-10-10

**Authors:** Alejandra Chavez-Ponce, Ahmed El Shaer, Sahar Samimi, Hasan Alarouri, Samian Sulaiman, Alyssa Harris, Mohamad Alkhouli

**Affiliations:** a Mayo Clinic, Rochester, Minnesota, USA; b Analyst Resource Team, Center for Advanced Analytics and Informatics, Chicago, Illinois, USA

Recent advances in transcatheter interventions refueled the interest in tricuspid regurgitation (TR). The focus has been on developing transcatheter treatment options for TR considering the substantial short-term mortality (∼10%) associated with surgical management of isolated TR.^1^ However, recent reports suggested that the adverse outcomes with isolated TR surgery may be mediated by the patient’s risk profile and late referral rather than by the surgery itself.^2^ We hypothesized that the growing interest in TR is associated with: 1) increasing utilization of surgical treatment for isolated TR; and 2) improvement in the outcomes of isolated TR surgery. We further speculated that such improvement is related to the changing risk profile of patients referred to surgery overtime. To test this hypothesis, we utilized a large consortium database (Vizient Clinical Data Base) to identify patients undergoing TR surgery between January 1, 2016 and December 31, 2021. The study was exempted by the institutional review board since the data utilized were deidentified. The Vizient Clinical Data Base has been utilized in studying various strategies for the management of valve disease in the United States.^3^

We identified 30,525 patients who underwent TR surgery during the study’s time frame using International Classification of Diseases-10th revision codes. We excluded patients who had concomitant cardiac surgery, infective endocarditis, and/or congenital tricuspid disease. We utilized the nonparametric Mann-Kendal test to assess for monotonic trends of in-hospital mortality after TR surgery. We performed a univariable and multivariable logistic regression utilizing the baseline characteristics variable to identify independent factors associated with in-hospital mortality.

**Figure 1:**
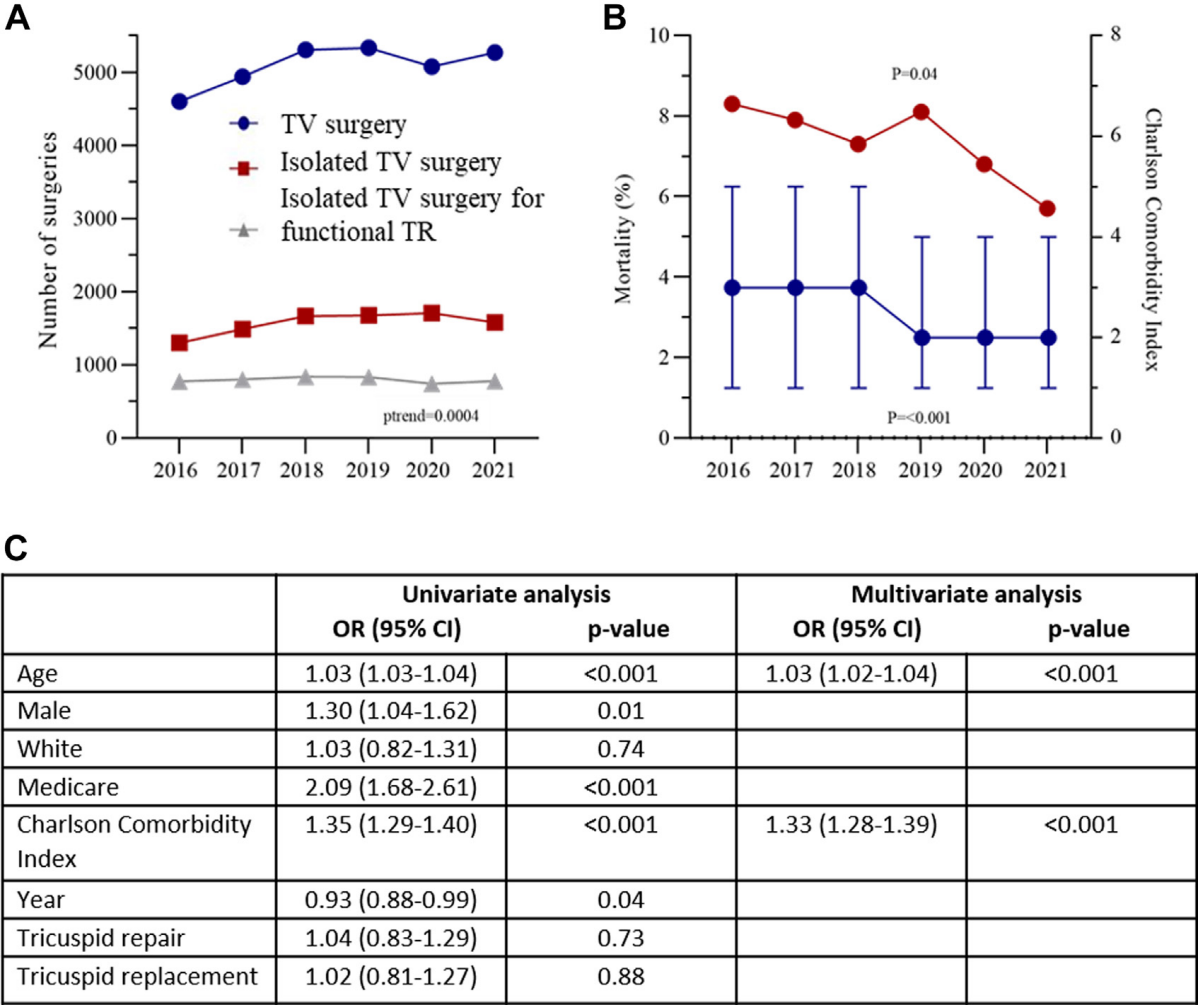
Evolving Trends in Tricuspid Surgery: Assessing Complexity and In-Hospital Mortality Rates (A) Temporal trends in the use of tricuspid valve surgery. (B) Temporal trends in the in-hospital mortality rate and Charlson comorbidity index following isolated tricuspid valve surgery. (C) Predictors of in-hospital mortality following isolated tricuspid surgery. TR = tricuspid regurgitation; TV = tricuspid valve.

A total of 4,753 patients who underwent isolated surgery for TR in 222 centers in the United States were included in the study. Mean age was 55.1 ± 16.4 years and 50.3% female. Among these patients, 49.4% underwent valve repair and 39.2% underwent valve replacement. Although total volumes for TR surgery increased overtime (*P* trend = 0.0004), surgery for isolated functional TR remained unchanged (Figure 1A). The prevalence of major comorbidities among patients referred to TR surgery decreased over time, as reflected in the Charlson comorbidity index (3.2 in 2016 vs 2.6 in 2021, *P* trend <0.001) (Figure 1B). In-hospital mortality occurred in 7.3% of the patients overall, but with significant improvement over the years (8.3% in 2016 vs 5.7% in 2021, *P* trend = 0.04). To identify factors associated with in-hospital mortality, we performed univariable and multivariable logistic regression. The models included demographics (age, sex, and insurance payer), comorbidity risk profile (Charlson comorbidity index), and year of surgery. In the univariable logistic regression analysis, the calendar year was associated with in-hospital mortality. However, in the multivariable backward stepwise regression model, only age (OR: 1.03; 95% CI: 1.02-1.04; *P* < 0.001) and Charlson comorbidity index (OR: 1.32; 95% CI: 1.27-1.38; *P* < 0.001) were independently associated with in-hospital mortality (Figure 1C).

The main findings of our focused analysis are: 1) surgical management of isolated functional TR remains infrequent in contemporary U.S. practice; and 2) the short-term outcomes of isolated TR surgery improved between 2016 and 2021 largely due to the changing risk profile of patients referred to surgery. Although speculative, the increasing availability of transcatheter TR therapies (repair and replacement) could have shifted the risk profile of patients referred to surgery such that lower-risk patients are now referred for surgery while higher-risk patients are treated with transcatheter therapies.^4^ As recently demonstrated by clinical trials such as TRILUMINATE PIVOTAL trial, transcatheter therapies have shown improvement in health status compared with medical therapy.^5^ If true, this hypothesis would further corroborate prior research suggesting that the historically suboptimal outcomes of surgery are more related to the delayed treatment, and the high risk profile of the patients referred to surgery than to the surgical intervention itself.^2^ It would also suggest a synergistic complementary impact of both transcatheter and surgical treatment of isolated TR overall.

This study has several limitations including its retrospective nature, the administrative nature of the database utilized, the variation in the database secondary to the number of hospital systems participating, and the lack of granular data on TR (disease duration, right ventricular function, pulmonary pressures, etc.). Importantly, this study does not address patients who were denied tricuspid valve surgery due to higher surgical risk nor those treated with transcatheter therapies. Despite these limitations, our study documents a promising trend toward further improvements in the outcomes of patients with severe isolated TR. Additional research utilizing clinical databases and incorporating the outcomes of medical and transcatheter management of TR can provide a holistic view of the optimal treatment of these patients.
